# Preparation of Anti-Human Podoplanin Monoclonal Antibody and its application in Immunohistochemical Diagnosis

**DOI:** 10.1038/s41598-018-28549-w

**Published:** 2018-07-05

**Authors:** Chengjie Xie, Rongzhi Wang, Abdullah F. U. H. Saeed, Qinghai Yang, Huiling Chen, Sumei Ling, Shiwei Xiao, Linmao Zeng, Shihua Wang

**Affiliations:** 1 Key Laboratory of Pathogenic Fungi and Mycotoxins of Fujian Province, Key Laboratory of Biopesticide and Chemical Biology of Education Ministry, and School of Life Sciences, Fujian Agriculture and Forestry University, Fuzhou, 350002 China; 2 Fuzhou Maixin Biotech.Co., Ltd, Fuzhou, 350100 China

## Abstract

Podoplanin (PDPN), a 38 kDa transmembrane sialoglycoprotein from human, is expressed in lymphatic endothelial cells but not in vascular endothelial cells, and has been considered as a specific marker of lymph. In this study, the gene encoding the extracellular part of PDPN (ePDPN) was synthesized and used to expressed fusion protein ePDPN-His and GST-ePDPN, respectively, in *E.coli*. The purified GST-ePDPN fusion protein was mixed with QuickAntibody-Mouse5W adjuvant to immune mice, and the antiserum titer was determined by indirect ELISA. A stable cell line named 5B3 generating anti-PDPN monoclonal antibody (mAb) was obtained by hybridoma technology. The isotype of 5B3 cell line was IgG_2b_, and the chromosome number was 102 ± 4. The 5B3 mAb was purified successfully from ascites fluid through Protein G column, and its affinity constant was 2.94 × 10^8^ L/mol. Besides, excellent specificity of the 5B3 mAb was further demonstrated in ELISA, western blot and immunohistochemistry experiments, suggesting that 5B3 mAb displays similar application value to D2-40, a commercial available antibody. Hence, the current study provides conclusive guidelines for preparation of other mAbs and their applications in immunohistochemistry diagnosis.

## Introduction

Podoplanin (PDPN) is an O-linked transmembrane sialoglycoprotein that consists of 162 amino acids with molecular weight 38 kDa^1–3^. Previous studies have shown that PDPN is a specific marker of lymphatic endothelial, and it is highly expressed in lymphatic endothelial cells but not in vascular endothelial cells^4^. PDPN can also be found in other tissues types, including follicular dendritic cells, reticular cells, mesothelial cells, testicular germ cells and ovarian cells^5^. Furthermore, PDPN has also been reported in lymphangiomas, Kaposi sarcomas, seminomas and epithelioid mesotheliomas hemangioblastomas^6^. To date, PDPN contributes to the diagnosis of lymphatic endothelial cell-derived tumors and to the judgement of lymphatic invasion and metastasis in other tumor tissues by labeling the lymphatic endothelium^7^. Therefore, it is important to develop methodologies for early diagnosis of lymphatic-related tumors using PDPN as a biomarker.

Currently, IHC detection has been recommended as gold standard for tumor diagnosis by the World Health Organization (WHO). Hence, the major challenge in the present study is generation of a monoclonal antibody with high affinity and specificity against PDPN for IHC diagnosis. To date, several commercial mAbs against PDPN have been used, including D2-40, NZ-1, 18H5, LpMab-9 and LpMab-10^8–10^. Among these mAbs, only D2-40 has been used for pathological diagnosis. However, dysgerminoma tissue as an antigen for producing the D2-40 faced challenges such as weak immune response by low concentration of PDPN protein, and many non-specific antibodies generated by numerous substances with the method^11^. In fact, it’s difficult for researchers to obtain carcinoma *in situ* like dysgerminoma tissue or to obtain cancer cell lines to immune mice. Furthermore, it’s challenging to select a stable eukaryotic cell line expressing PDPN since the cells can be contaminated during an extended cell culturing process. In view to the current situation, it is urgent to establish a rapid, easy, cost-effective, feasible and reliable method such as expressing the PDPN antigen in *E. coli*, and producing a novel anti-PDPN mAb based on the resulted antigen to diagnose related tumors by IHC. The present study provides insights into establishing new methodologies for rapid preparation of such novel mAbs.

## Materials and Methods

### Reagents

All the strains used in this study were from Fujian Agriculture and Forestry University (Fujian, China). The anti-His 6 tag mAb, anti-GST tag mAb and horseradish peroxidase (HRP) labeled goat anti-mouse IgG were purchased from ZSGB-Bio Co., Ltd (Beijing, China). D2-40 antibody and all paraffin-fixed tissue sections were supported by Fuzhou Maixin Biotech Co., Ltd (Fuzhou, China). Hypersensitivity ECL chemiluminescence detection kit was purchased from Wuhan Sanying Co., Ltd (Wuhan, China). Hypoxanthine, aminopterin and thymidine supplement (HAT), hypoxanthine and thymidine supplement (HT) and polyethylene glycol 1450 solution (PEG 1450) were purchased from Sigma. RPMI medium 1640 powder and fetal bovine serum (FBS) were purchased from Gibco.

### Mice and QuickAntibody-Mouse5W adjuvant

Female *Balb/c* mice (6 to 8 weeks old) were obtained from Wushi Animal Laboratory (Shanghai, China). All animal experiments were performed according to the protocols approved by the Animal Ethics Committee of the Fujian Agriculture and Forestry University. QuickAntibody-Mouse5W adjuvant (Quickadjuvant) was purchased from Beijing Biodragon Immunotechnologies Co., Ltd (Beijing, China).

### Cells

Murine myeloma cell line SP2/0 from our laboratory was cultured in RPMI-1640 medium with 10% FBS. Hybridoma cells were cultured in RPMI-1640 medium with 20% FBS containing HAT. After 10 d of cell fusion, hybridoma cells were cultured in RPMI-1640 medium with 20% FBS containing HT, and screened by indirect ELISA (iELISA) to obtain a stable cell line that stably secreting mAb against PDPN. The selected hybridoma cells were further expanded in RPMI-1640 medium with 10% FBS.

### Codon optimization and synthesis of ePDPN gene

The identifier of PDPN in the Uniprot is Q86YL7. According to the database, the amino acids of 23–131 is noted as the extracellular part of PDPN (ePDPN) and was selected for codon optimization for expression in *E. coli* Transetta (DE3) and evaluated by graphical codon usage analyser (http://gcua.schoedl.de)^12^. The optimized DNA sequence of ePDPN was synthesized by Shanghai BioSune Biotech Co., Ltd (Shanghai, China).

### Expression and purification of ePDPN antigen

After DNA amplification by PCR, the resulted products were digested by *Nco* I/*Xho* I and *EcoR* I/*Not* I, respectively, and inserted into plasmids of pET-28a and pGEX-6P1 digested with corresponding enzymes^13^. More information about primers was detailed in Table 1. The ligated DNA mixture was transformed into the *E. coli* Transetta (DE3) competent cells by calcium chloride transformation. After verification by PCR and sequence alignment, target protein expression was induced by addition of isopropyl-β-d-thiogalactoside (IPTG).

**Table 1:** Primers for PCR.

Name	Primer Sequence
28a-*Nco* I	5′-TTACCATGGCGAGCACCGGCCAAC-3′
28a-*Xho* I	5′-AAACTCGAGCAGGGTTACGGTACTCAAG-3′
6P1-*EcoR* I	5′-TCCGAATTCATGGCGAGCACCGGCCAACC -3′
6P1-*Not* I	5′-GTTGCGGCCGCTTACTCGAGCAGGGTTACG -3′

The cell culture was harvested at 4 °C by centrifugation at 6,000 r/min for 5 min. The pellets were resuspended with buffer A (0.01 mol/L imidazole buffer, pH 8.0) and the cells were broken by sonication. The supernatant after centrifugation at 12,000 r/min for 10 min was collected and incubated for 30 min at 4 °C with Ni^2+^ affinity resin in a buffer A. Pre-chilled 0.02 mol/L imidazole buffer (pH 8.0) was used to wash the resin to remove substances that bound non-specifically. The target protein was gradually eluted by adding pre-cooled 0.25 mol/L imidazole buffer (pH 8.0).

The procedure for GST-ePDPN purification was similar to that of purification of ePDPN-His protein. GST fusion protein purification resin was used to incubate with collected supernatant after sonication containing the GST-ePDPN. Phosphate-buffered saline (PBS, 0.01 mol/L) was used to wash the resin to reduce binding of non-specific proteins. After washing, the target protein GST-ePDPN was eluted by GST elution buffer (0.01 mol/L reduced glutathione dissolved in 0.05 mol/L Tris-cl solution, pH 8.0). The concentration of ePDPN-His and GST-ePDPN fusion proteins was determined by BCA method and Nanodrop (Thermo), respectively^14^.

### Identification of fusion ePDPN antigen

Both purified recombinant ePDPN fusion proteins were identified by western blot^15^. Protein loading buffer was added into tubes containing protein sample and the mixture was incubated in boiling water for 10 min. The sample was centrifuged at 12,000 r/min for 5 min, and the supernatant was collected for running SDS-PAGE. The proteins on gel were transferred to a PVDF membrane and blocked by PBS with 5% milk (5% PBSM) at room temperature for 1 h. After washing three times with PBS, anti-His 6 tag mAb and anti-GST tag mAb (at dilution of 1:8000) was added into the reaction and incubated at room temperature for 1 h followed by washing of the PVDF membranes three times with PBS containing tween-20 (PBST) and three times of PBS. Subsequently, HRP-labeled goat anti-mouse IgG (diluted at 1:8000 with 5% PBSM) was incubated with the reaction mixture at room temperature for 1 h. After washing three times with PBST and PBS, both PVDF membranes were incubated with mixture of ECL chemiluminescence reagents followed by imaging in fully functional multicolor fluorescence imaging instrument (Bio-Rad).

### Animal immunization and titer analysis by iELISA

Three female *Balb/c* mice (6 to 8 weeks old) were injected intra-muscularly with 60 μg of GST-ePDPN fusion protein appropriately mixed with 150 μL Quickadjuvant (final volume adjusted to 300 μL with 0.9% saline solution) as first immunization. Subsequent injections were given at intervals of 20 days, respectively. Blood was extracted from the tail vein of each mouse after third immunization, and the titer was determined by iELISA^16^. Briefly, 100 μL of ePDPN-His fusion protein as coating antigen diluted to 5 μg**/**mL with carbonate/bicarbonate coating buffer was added into each well of the ELISA plate, and incubated at 37 °C for 2 h. Plate was washed and blocked with 5% PBSM (200 μL/well), and incubated at 37 °C for 2 h. Then, 100 μL of serially diluted anti-PDPN antiserum was added into each well followed by incubation at 37 °C for 1 h. After washing properly, HRP-labeled goat anti-mouse IgG (diluted at 1:8000 in 5% PBSM, 100 μL/well) was added and incubated at 37 °C for 1 h. Plate was washed and 100 μL substrate solution was added into each well and incubated at 37 °C for 10 min. Reaction was stopped by adding 2 mol/L H_2_SO_4_ (50 μL/well), and the optical density (OD) value at 450 nm was determined by micro-plate reader (Thermo).

### Cell fusion and hybridoma screening

The mouse that showed highest titer was injected intraperitoneally with 20 μg GST-ePDPN antigen mixed with 100 μL 0.9% saline solution to boost immunization. Three days later, splenocytes from the immunized mouse were mixed with murine SP2/0 myeloma cells at a ratio of 10:1 in the presence of 1 mL PEG 1450 to generate hybridoma cells. Subsequently, RPMI-1640 mixed with 20% FBS/HAT medium containing hybridoma cells was equally distributed (200 μL/well) into 96-well micro-titer cell culture plates in which feeder cells have been added a day before cell fusion, and incubated at 37 °C with 5% CO_2_^17^. After 5 d, half of the supernatant in each well of micro-titer plates was substituted with fresh medium. Ten days after cell fusion, the titer of the supernatant of culture medium was determined by iELISA for anti-PDPN mAb activity. A stable cell line was obtained by subsequent limiting dilution method until the positive percentage reached 100%.

### Characterization of positive hybridoma cells against PDPN

To analyze the mAb isotype, ELISA was performed following the instruction of mouse mAb Isotyping (IgA, IgM, IgG_1_, IgG_2a_, IgG_2b_, IgG_3_) Kit^18^. Chromosome analysis was carried out according to previously published protocols from our lab^19^. Hybridoma cells on glass slides were stained with Giemsa staining solution, and the number of chromosomes in the hybridoma cell with distinct dispersion was counted under microscope.

### Production of anti-PDPN mAb

To obtain large amount of mAbs and to reduce possibilities of cell contamination, the ascites method was selected. *Balb*/c mouse was injected intraperitoneally with 0.5 mL paraffin oil. One week later, about 1 × 10^6^ positive hybridoma cells were injected into mouse intraperitoneally. The ascites fluid was collected through a needle after at least one week. After centrifugation, the middle layer of the ascites fluid containing mAb was carefully transferred into a new tube followed by purification with Protein G, and the purified mAb was analyzed by SDS-PAGE^20^. The concentration of the purified mAb was determined under the manufacturer instruction of BCA protein assay. The titer of the mAb was measured by iELISA with similar protocol used in the experiment of titer analysis described above, and negative control was ascites from negative mouse.

### Affinity determination of mAb

The affinity of mAb against PDPN was determined followed procedures previously published^21^. After coating with different concentrations of ePDPN-His (4, 1.5 and 0.5 μg/mL), wells were added with serially diluted anti-PDPN mAb followed by adding goat anti-mouse HRP-IgG and substrate. The OD value of each well was measured at 450 nm by micro-plate reader after the reaction was stopped. A curve diagram based on equation (1) was developed to show the relationship between concentration of the antibody as the abscissa and the value of absorption as the ordinate. Relative affinity of anti-PDPN mAb was measured in pairs according to formula (2), where [Ab] or [Ab]_t_ was from x_0_ of Table 2^22^. The average of their affinity was used as the final result.

1 $$y={A}_{2}+\frac{{A}_{1}-{A}_{2}}{1+{(\frac{x}{{x}_{0}})}^{p}}$$

2 $$K{\rm{a}}=\frac{{\rm{n}}-1}{2\times (n{[Ab]}_{t}-[Ab])}$$

[Ab] is the concentration of antibody at the 50% inhibition of control values (IC_50_) when the concentration of antigen is [Ag]; [Ab]_t_ is the concentration of antibody at IC_50_ when the concentration of antigen is [Ag]_t_; and n represents the ratio of [Ag] to [Ag]_t_.

**Table 2:** Parameters of fitting curve based on equation (1).

Concentration (ng/mL)	Parameter	Value	Standard Error	Adj. R-Square
4000	x_0_	6.51544	9.47014	0.99147
	A_1_	−2.64599	3.63223	
	A_2_	3.62507	0.20052	
	p	0.73334	0.25921	
1500	x_0_	14287.891	5.81974E9	0.975456
	A_1_	−3.38706	41.95306	
	A_2_	21.54778	744.10623	
	p	0.11967	1.71155	
500	x_0_	224.28169	34.01063	0.99544
	A_1_	0.07755	0.03482	
	A_2_	1.62498	0.12436	
	p	1.21719	0.17486	

### Stability analysis of mAb by IHC

To analyze stability of the antibody, the anti-PDPN mAb was incubated at 37 °C or 4 °C for several days followed by analysis by IHC method. After dewaxing and hydration, sections from paraffin-fixed normal rectal tissues were heated under high pressure with 10 mM citrate buffer (pH 6.0) to repair antigen. Then, sections were washed three times with PBS. After blocking by 3% H_2_O_2_ for 10 min and washing by PBS, the mAb was added into the sections and incubated for 1 h followed by washing. MaxVisionTM/HRP reagent was introduced into the sections and incubated for 25 min. After washing by PBS, the 3,3-diaminobenzidine (DAB) chromogenic liquid was added into the sections and incubated for 10 min followed by stopping with water. After redyeing with hematoxylin for 25 s and returning to blue for 30 s, the sections were dehydrated by gradient of ethanol, and treated by xylene for 3 min. Finally, the sections were sealed by neutral gum and checked under microscope.

### Determination of specificity of mAb

The specificity of mAb against PDPN was determined by three methods described as follow. In the experiment of iELISA, ePDPN-His, His-B-cell lymphoma 6 protein (His-Bcl6), His-Chromograin A (His-CgA), Albumin Human (HSA) and Interferon-gamma (IFN-γ) from human as coating antigens were diluted to 5 μg/mL and added into the micro-titer plates (100 μL/well), respectively. After anti-PDPN mAb (at a dilution of 1:8000) was added, goat anti-mouse HRP conjugate and substrate were added subsequently, the reaction was stopped and the absorbance of each well was measured for analysis. The second method to identify the specificity of the mAb was western blot. After respectively running in 15% and 10% SDS-PAGE gel, ePDPN-His and GST-ePDPN fusion proteins were transferred into the PVDF membrane and subsequently blocked. The membrane was incubated with the mAb at a dilution of 1:8000 and then goat anti-mouse HRP-IgG. ECL chemiluminescence detection reagents were mixed and added, and imaging was carried out in fully functional multicolor fluorescence imaging instrument. The protocol of IHC was the same with that of stability analysis of mAb against PDPN described above. After sections from lung tissue, mesothelioma, seminoma, submucosal lymphatic vessels and thyroid were interacted with anti-PDPN mAb, MaxVisionTM/HRP reagent and the DAB chromogenic liquid were added subsequently. The sections were treated with a series of chemicals followed by observation and analysis.

## Results

### Verification and synthesis of the PDPN sequence after optimization

**Figure 1:**
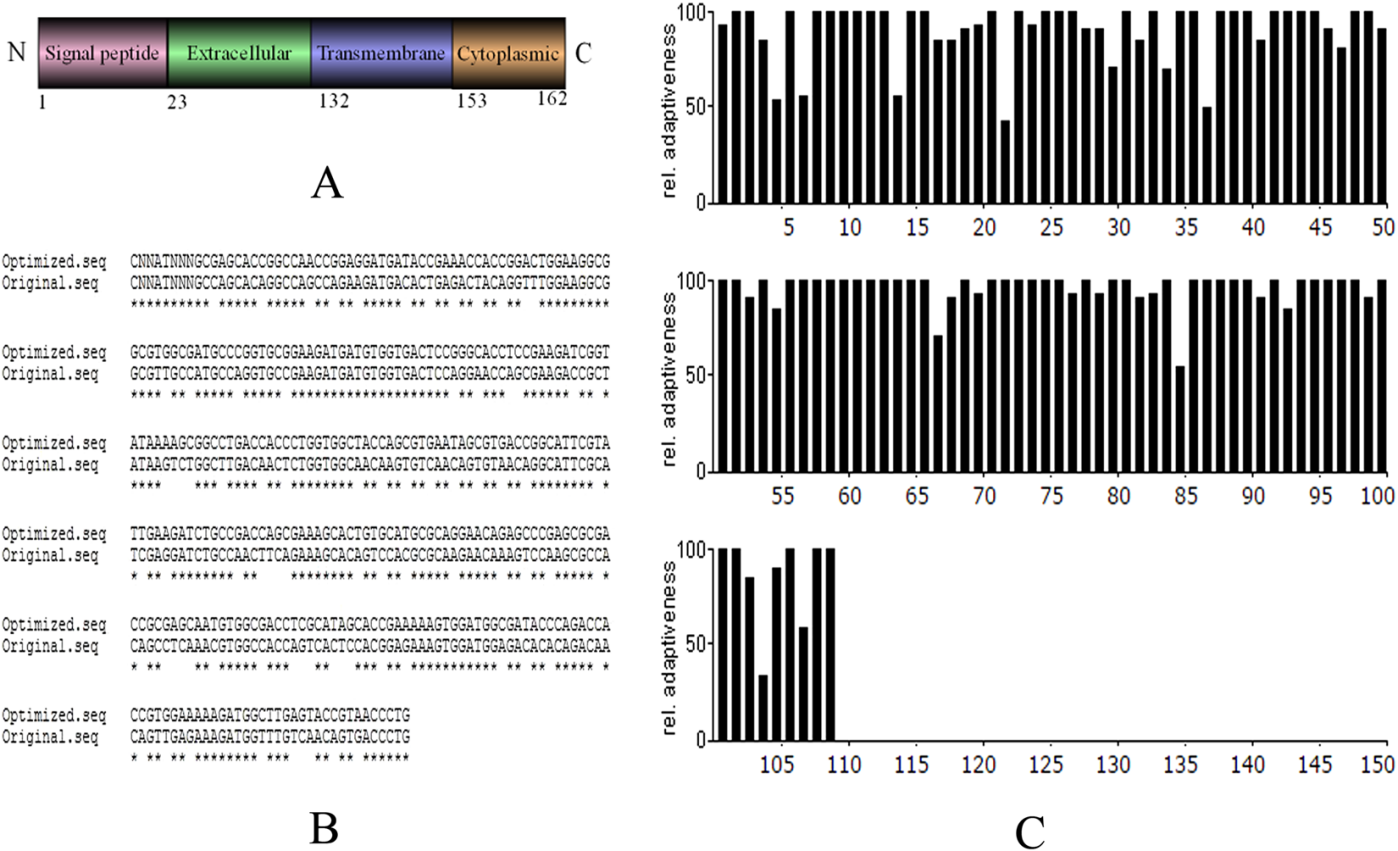
Analysis of PDPN structure and sequence alignment of e*PDPN* (the extracellular part of PDPN) after optimization. (**A**) Analysis of PDPN structure. (**B**) DNA sequence alignment of e*PDPN* between original and optimized sequences. (**C**) Identification of the optimized sequence for e*PDPN* by graphical codon usage analyser (http://gcua.schoedl.de).

Molecular structure of PDPN was divided into four parts according to the Uniprot, including signal peptide, extracellular part, transmembrane part and intracellular part (Fig. 1A). Since PDPN is a transmembrane protein, the extracellular domain is the main part for generating antibodies for diagnostic or other applications. Also, considering the likelihood of solubility of antigen expressed in *E. coli*, the ePDPN which has a better chance of being soluble than the intact membrane protein was used for antigen generation. The human ePDPN DNA sequence needs to be codon optimized for *E. coli* expression, and Fig. 1B showed comparison between original and optimized sequences, both of which encode exactly same protein sequence (Fig. S1). The optimized codon was identified by graphical codon usage analyzer (http://gcua.schoedl.de) that the relative adaptiveness of all codons was more than 20% meaning all the codons should be suitable for expression in *E. coli* Transetta (DE3) **(**Fig. 1C). The optimized DNA sequence of ePDPN was synthesized by Shanghai BioSune Biotech Co., Ltd (Shanghai, China).

### Expression and identification of ePDPN antigen

**Figure 2:**
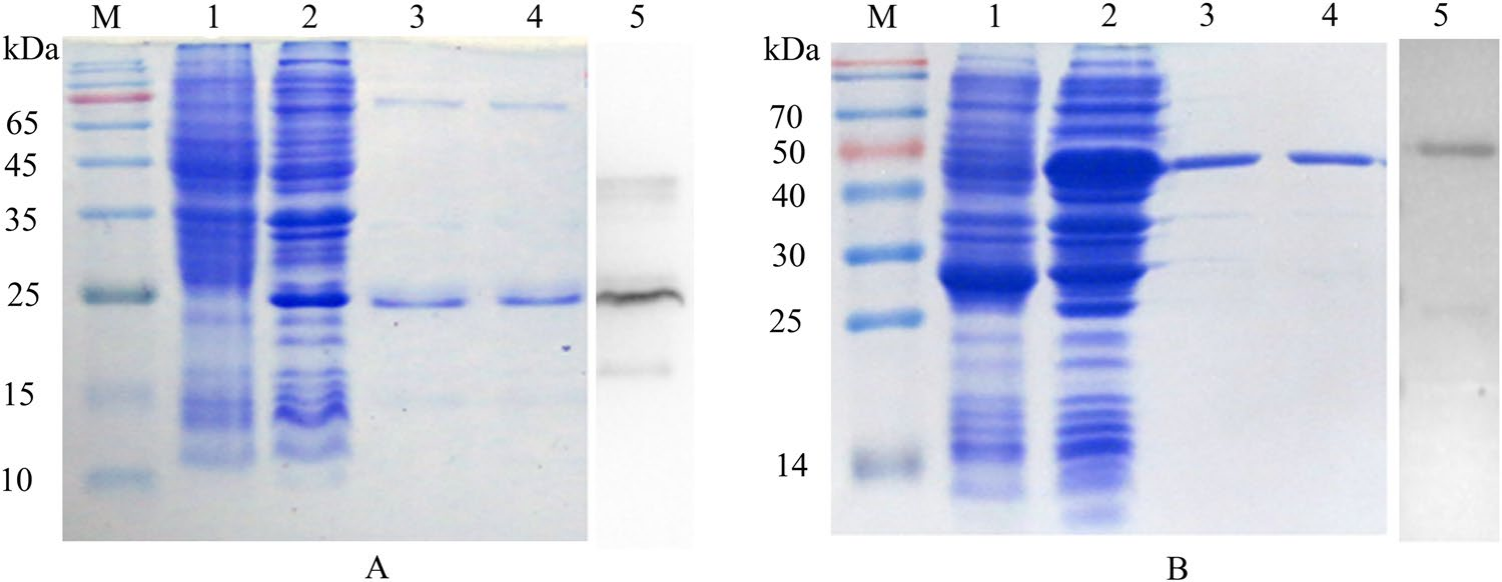
Expression and identification of recombinant ePDPN fusion proteins. (**A**) Purification and identification of ePDPN-His. Lane M: Markers; lane 1: empty vector of pET-28a; lane 2: total protein of pET-28a-PDPN in *E. coli* Transetta (DE3); lane 3–4: purified ePDPN-His; lane 5: western blot results of ePDPN-His reacted with anti-His 6 tag mAb; (**B**) Purification and identification of GST-ePDPN. Lane M: Markers; lane 1: empty vector of pGEX-6P1; lane 2: total protein of pGEX-6P1-ePDPN in *E. coli* Transetta (DE3); lane 3–4: purified GST-ePDPN; lane 5: western blot results of GST-ePDPN reacted with anti-GST tag mAb.

The amplified DNA fragment of ePDPN was inserted into vectors pET-28a and pGEX-6P1, respectively, for expression in *E. coli* Transetta (DE_3_). After purification, the obtained fusion proteins of ePDPN-His and GST-ePDPN were identified by SDS-PAGE and western blot. Compared to empty vector, specific distinct bands were observed at 24 kDa (ePDPN-His) and at 50 kDa (GST-ePDPN) after IPTG induction and affinity chromatography (lane 1~4 in Fig. 2). The concentration of ePDPN-His and GST-ePDPN was determined to 1.7 mg/mL and 0.3 mg/mL, respectively. Both of target proteins were recognized by their respective labeled antibodies (lane 5 in Fig. 2), and further indicating that the production of ePDPN was successful.

### Immunization and screening of positive hybridoma clones

**Figure 3:**
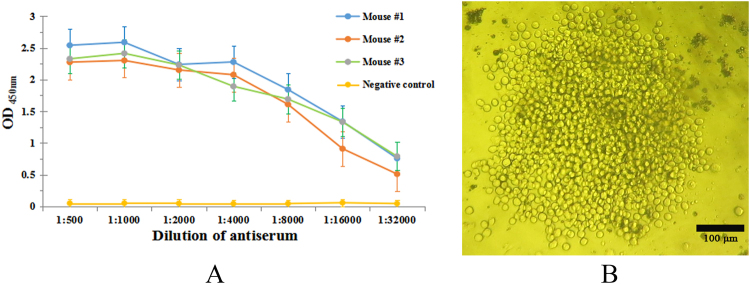
Titer determination of mice antiserum and observation of hybridoma cells. (**A**) Titer determination of mice antiserum by iELISA. Two replicates were performed in each test. (**B**) The observation of hybridoma cells cultured in HAT medium. Hybridoma cells were checked after 9 d of fusion (X40).

GST-ePDPN recombinant protein was injected to immune female *Balb/c* mice, and ePDPN-His protein was used as the coating antigen. After immunizations four times, the titer of antiserum from mouse were determined by iELISA. The ELISA showed that the titer from immunized mice were significantly higher than that from the negative control (Fig. 3A). The results further indicated that the immunization of mouse with GST-ePDPN fusion protein successfully induced immunogenicity for subsequent production of mAb against PDPN. Therefore, the mouse was selected for cell fusion. Spleen cells from the above immunized mouse were harvested aseptically and fused with SP2/0 myeloma cells at the ratio of 10:1 in the presence of PEG 1450 as the fusion reagent. After the fusion reaction, feeder cells, spleen cells, unfused myeloma cells and fused cells originated from only myeloma cells or from only spleen cells could not grow in HAT selection medium. Whereas, hybridoma cells formed by fusion of spleen cells and myeloma cells should grow successfully in the medium. On the first day of fusion, all types of cells were observed under inverted microscope. Three days later, few small hybridoma cell clones appeared to grow in the medium. As the cells proliferated, the scale of the growing cell clones became larger and larger, and they reached to a confluence covering 70% of the area at the bottom of the 96-well plates after 9 d of fusion (Fig. 3B). In this study, hybridoma cell clones from 9 wells out of 672 wells showed positive activity of anti-PDPN mAb determined by iELISA with the positive clone rate of 1.3%. The nine hybridoma cell clones were named 2F9, 3E1, 4D1, 4E12, 4F12, 5B3, 7C5, 7A11 and 7D11, respectively. Among them, the 5B3 clone showed highly significant specificity in ELISA and IHC from the initial screening. More information is described later in this article, and the 5B3 hybridoma cell line was selected for further experiments.

### Identification of hybridoma cells

**Figure 4:**
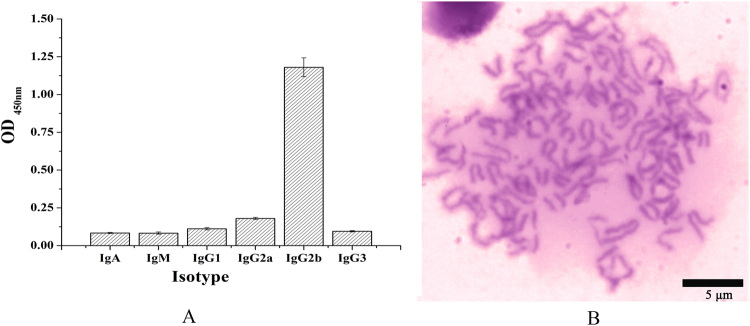
Identification of hybridoma cell line 5B3. (**A**) The isotype of anti-PDPN hybridoma cell line 5B3. The isotype of mAb 5B3 was determined by isotyping (IgG_1_, IgG_2a_, IgG_2b_, IgG_3_, IgA, IgM) kit, and the isotype of 5B3 is IgG_2b_. (**B**) Chromosome analysis of 5B3 hybridoma cell (X400). The chromosome number of 5B3 cell line was 102 ± 4 after counting ten cells (repeat 3 times/cell), demonstrating that the 5B3 cell line was successfully produced by fusion of spleen cells and myeloma cells.

To identify antibody isotype of the anti-PDPN mAb produced from the 5B3 cell line, a capture ELISA method was performed by using isotyping mAb kit. The results showed that the isotype of 5B3 mAb is IgG_2b_ (Fig. 4A), the isotype that can be purified by Protein G column and with the longest half-life of about 23 days among all the immunoglobulins. The 5B3 cell line was also used for the follow-up tests. To analyze chromosomes, the 5B3 cells were fixed on a glass slide, stained by Giemsa cell staining solution, and observed under inverted microscope. The results showed that the chromosome number of 5B3 cell line was 102 ± 4 (Fig. 4B), suggesting that the chromosome number of the hybridoma cell 5B3 was approximately equal to the total chromosome number of spleen cell (39 ± 1) and myeloma cell SP2/0 (66 ± 4). The results further demonstrated that the 5B3 cell line was produced by the successful fusion of spleen cells and myeloma cells.

### Purification of monoclonal antibody from ascites fluid

**Figure 5:**
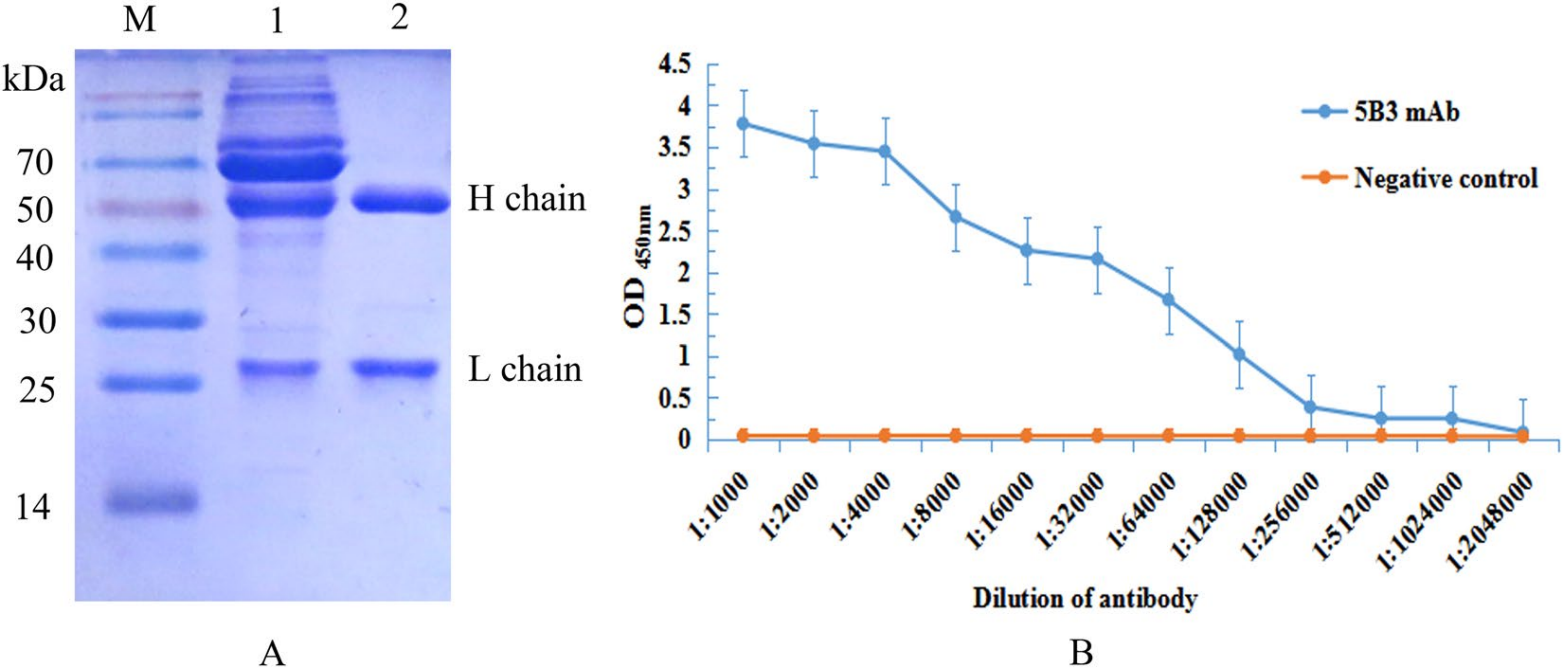
Analysis of purified mAb. (**A**) SDS-PAGE analysis of the purified mAb. Lane M: Marker, lane 1: ascites fluid, lane 2: the purified 5B3 mAb. (**B**) Titer of 5B3 mAb was determined by iELISA. The minimum OD value greater than 1 is at a titer of 1.28 × 10^5^ in this figure, indicating that the antibody remained active after purification. Negative control was ascites from negative mouse. Two replicates were performed for each test.

The 5B3 hybridoma cells were injected into the peritoneal cavity of pristine-primed *Balb/c* mice for producing ascites fluid, and the resulted ascites fluid was collected and purified by affinity chromatography using Protein G columns. SDS-PAGE was performed to analyze the purified mAb. The results showed two distinct bands with the heavy chain at 50 kDa and the light chain at 26 kDa (Fig. 5A), indicating that 5B3 mAb was successfully purified. After determination by BCA protein assay, concentration of the purified 5B3 mAb was 0.6 mg/mL. The titer of the mAb was determined by iELISA, and the results showed that the titer of anti-PDPN mAb reached 1.28 × 10^5^ (Fig. 5B), demonstrating that the mAb remained active after purification and could be used for further characterization.

### Characterization of the anti-PDPN monoclonal antibody

**Figure 6:**
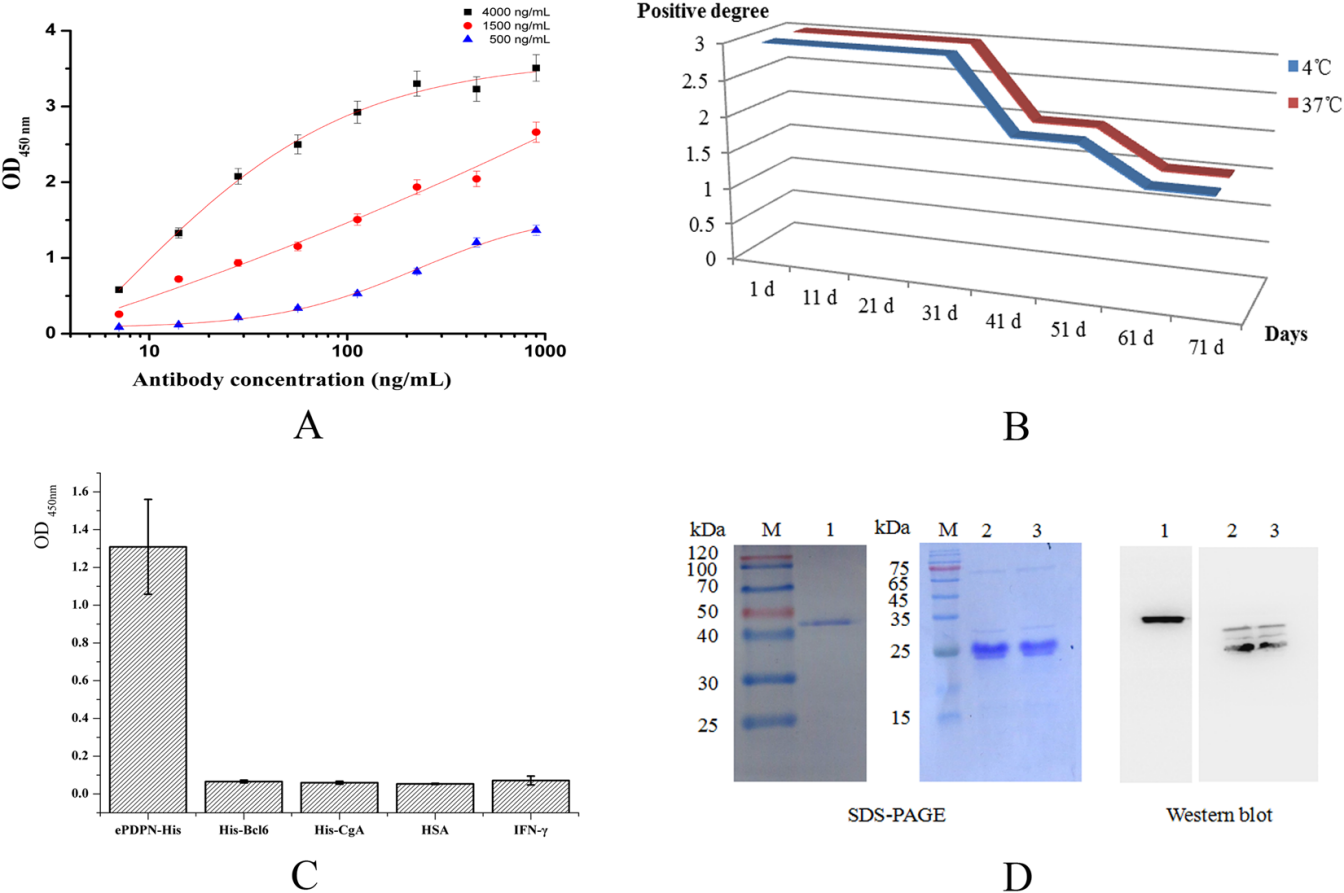
Characterization of purified 5B3 mAb. (**A**) Affinity was analyzed by iELISA. Different concentrations (4, 1.5 and 0.5 μg/mL) of coating antigen (ePDPN-His) were used to determine [Ab] and [Ab]_t_ in the formula^2^ that were from x_0_ in Table 2, and the average affinity was 2.94 × 10^8^ L/mol. Two replicates were performed in each test. (**B**) Stability of the 5B3 mAb against PDPN was determined by IHC. The results demonstrated that the mAb maintained good stability in tested temperature. “3” on the Y axis indicated strong positive, “2” on the Y axis indicated normal positive, and “1” on the Y axis indicated weak positive. Each test was performed two replicates. (**C**) The specificity of the 5B3 mAb was determined by iELISA. Two replicates were performed in each test. (**D**) The specificity of the 5B3 mAb was determined by western blot. Lane M: Protein Marker, Lane 1: GST-ePDPN, Lane 2, 3: ePDPN-His.

The affinity analysis of 5B3 mAb was performed by iELISA, and the parameters of the fitting curves (Fig. 6A) based on equation (1) was shown in Table 2. The results showed that the affinity of 5B3 mAb was 2.94 × 10^8^ L/mol, suggesting that the 5B3 mAb was sensitive to PDPN. To further analyze the stability, the 5B3 mAb was incubated at 37 °C and 4 °C then dropped into sections from normal rectal tissues at different days, respectively. The results showed that the mAb remained high sensitivity within 31 days, and maintained normal sensitivity at 37 °C for two months. The results indicated that the purified 5B3 mAb has a significant stability (Fig. 6B). The specificity analysis of the 5B3 mAb was determined by iELISA, western blot and IHC. The iELISA results showed that the 5B3 mAb has high specificity in response to ePDPN-His and no cross-reactivity to the other related antigens was observed (Fig. 6C). Western blot results exhibited two distinct bands, GST-ePDPN in lane 1 and ePDPN-His in lane 2, 3 (Fig. 6D), and other bands were multimers of His-ePDPN and degradants of the protein trimer or tetramer, suggesting that the 5B3 mAb specifically recognized the target proteins GST-ePDPN and ePDPN-His. The results further demonstrated that the 5B3 mAb is highly specific to PDPN.

### Tissue sections detection for the monoclonal antibody against PDPN

**Figure 7:**
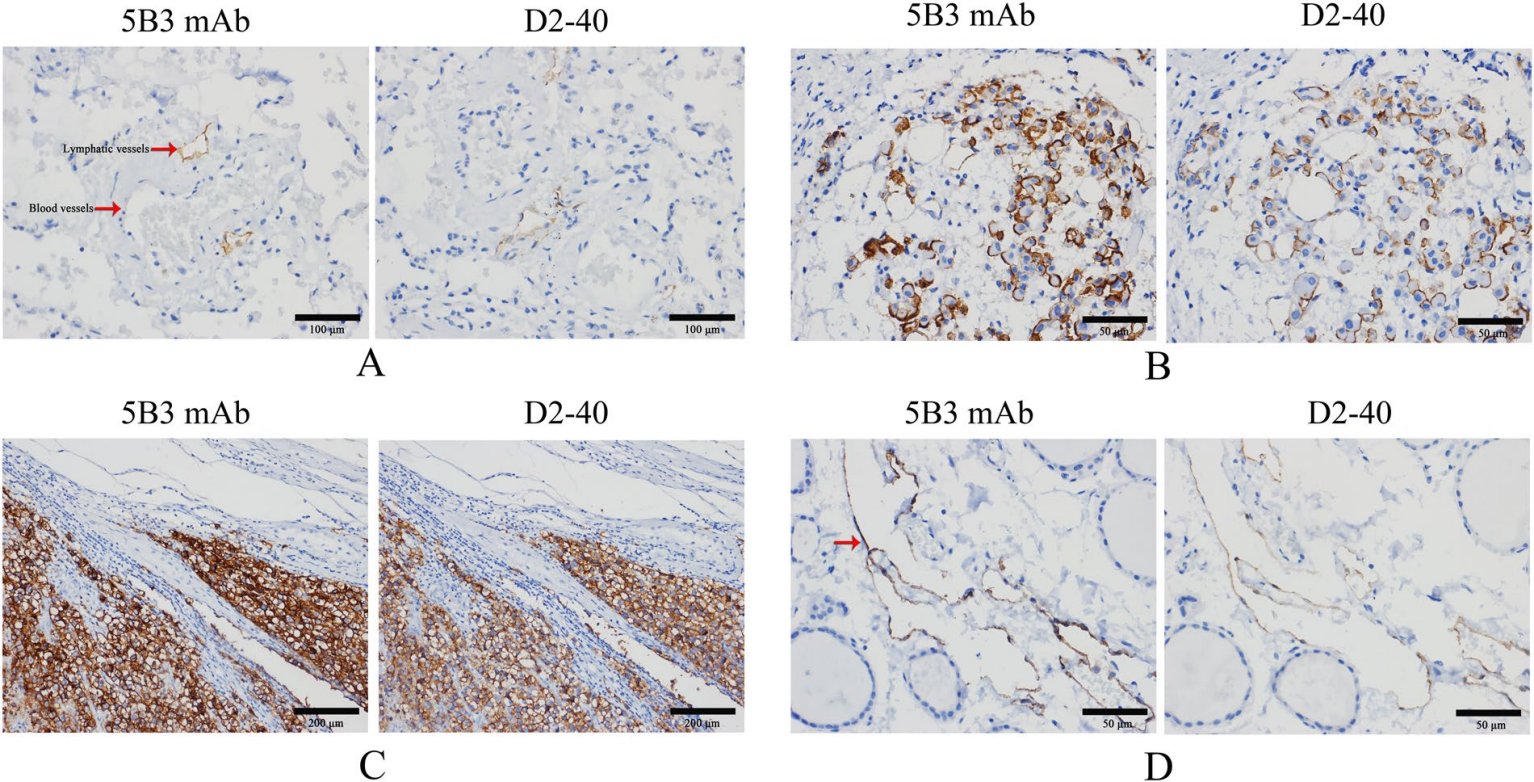
The specificity of the 5B3 mAb. The 5B3 mAb was analyzed in lung tissue (**A**) (X200), in mesothelioma (**B**) (X400), in seminoma (**C**) (X100) and in thyroid (**D**) (X400) by IHC. The sections in left were added the 5B3 mAb, and the sections in right were added the D2-40 mAb. Left and right arrows point to the blood vessels and lymphatic vessels in Fig. 7A, and the one in Fig. 7D displayed non-specific staining of smooth muscle in thyroid. Two replicates were performed in each test.

All the staining results of tissue sections dropped 5B3 mAb were highly similar to the positive control dropped D2-40 (Fig. 7). 5B3 mAb specifically recognized the lymphatic vessels and did not recognize any blood vessels in the lung tissue (Fig. 7A) and submucosal lymphatic vessels (not shown). The mesothelioma cells were stained in the membrane, and the staining degree in experimental group was much stronger than that of the positive group (Fig. 7B). Tumor cells were also stained in the sections of spermatogonia (Fig. 7C), suggesting that PDPN was expressed in normal cells of spermatogonia. However, extremely weak non-specific staining of smooth muscle was observed, although the whole staining was same to the positive control in thyroid (Fig. 7D). These results suggested that the specificity of the 5B3 mAb is highly similar to D2-40 except in smooth muscle. The results also demonstrated that our approach to prepare an IHC antibody is feasible and reliable.

## Discussion

Although some mAbs against human podoplanin have been produced and are now commercially available, the method and process of McAb preparation described in this study were different with traditional methods. Procurement of the encoding gene of PDPN was the first problem we encountered. Currently, several strategies are available, such as extracting DNA from paraffin wax, extracting RNA from cells, and gene synthesis. Expressing genes in eukaryotic cells needs introns, and may usually face difficulties of obtaining tumor tissues and cell lines that profoundly expressing PDPN. Gene synthesis method has many advantages such as cost-effectiveness, time efficient and sequence accuracy. Therefore, gene synthesis was selected. Meanwhile, it’s important for researchers to consider the codon bias between homo sapiens and *E. coli* for successful expression of the transmembrane protein. The ePDPN was selected for further immune response generator since it is exposed physiologically for antibody access. Graphical codon usage analyser was used for the identification after codon optimization, and the relative adaptiveness of all codon was more than 20%, indicating the optimized codons are likely to facilitate expression of ePDPN in *E.coli*.

It has been reported that the human ciliary neurotrophic factor with 6-His tag showed soluble expression after optimization under all test conditions^23^. In fact, it’s the first report regarding using recombinant GST-fused PDPN with codon optimization to prepare a monoclonal antibody against PDPN for immunohistochemical diagnosis, and the method used in this study is more efficient than those traditional methods^22^. Compared to the dysgerminoma tissue immunization, the highly pure PDPN antigen used in this study possess better chances of triggering an immune response, and reducing non-specific antibodies generation, further enhancing the efficiency of screening of monoclonal antibody with high affinity and specificity. Previously published paper showed that the GST-fused recombinant proteins were expressed successfully at high soluble levels in *E.coli* after codon optimization, including VP1, VP2, and VP3 of Chinese sacbrood virus. High antibody levels and lymphocyte proliferation were induced and promoted via immunization with the purified GST-tag fusion protein^24^. Our results are consistent with the finings described in references above.

The observation that the ePDPN exhibits different oligomeric states (multimer/monomer) may indicate that the natural PDPN protein may perform biological functions in multimer forms. Dimer may be the most stable structure for PDPN, since it is resistant to dissociation under SDS treatment (Fig. 6D). Similar phenomenon was also observed in our previous experiments (data not shown). Another lighter band showed on Fig. 6D may be multimer of His-ePDPN or degradation products from unstable trimer or tetramer of His-ePDPN. For GST-ePDPN, aggregation of the ePDPN protein may be prevented by GST protein fusion, or by folding effects driven by fusion with GST protein.

In the early stage of the study, Freund’s adjuvant was used to mix with GST-PDPN fusion protein to immunize *Balb/c* mice for immunogenic response. However, several hybridoma cells produced by fusing spleen cells from the mice and myeloma cells could not recognize PDPN in sections from paraffin-fixed normal rectal tissues even after six times of cell fusion. Unlike Freund’s adjuvant, Quickadjuvant is less likely to destroy the structure of antigen during mixing, and required no emulsification. Additionally, inflammation in the mice by intramuscular injection was not observed. Furthermore, antibody that recognizes a conformational epitope could be easily obtained from mice immunized with Quickadjuvant^25^. It has been previously reported that mixing of Alteplase antigen with Quickadjuvant produced a specific antibody against recombinant human plasminogen activator^26^. Therefore, Quickadjuvant was used to inject three mice in the study, and mAb cell line 5B3 was produced from these mice after cell fusion for three times. The antibody showed specific binding with PDPN.

Compared to other commercial antibodies, only D2-40 antibody against PDPN has been used for IHC diagnosis in the market to date. In this study, the positive stable hybridoma cell line 5B3 secreting mAb against PDPN was obtained, and the anti-PDPN mAb was purified from ascites with the titer of 1.28 × 10^5^. In addition, the 5B3 mAb kept a similarly high stability within 31 days after incubation at 4 °C and 37 °C. Moreover, the isotype of 5B3 mAb is IgG_2b_, a stable and widely used isotype for mAb. The affinity of 5B3 mAb is 2.94 × 10^8^ L/mol, while the affinity of D2-40 was not disclosed, suggesting that 5B3 mAb is a high affinity mAb^27^. 5B3 mAb showed great opportunities for wider applications as it exhibited affinity range from 10^7^ to 10^12^ L/mol^28^. The specificity of the 5B3 mAb was analyzed by iELISA, western blot and IHC. The results showed that 5B3 mAb has an exceptional specificity for recognizing PDPN, especially in sections from lung tissue, mesothelioma, seminoma and submucosal lymphatic vessels. The results are in consistency with the IHC results of reported organization tissue sections previously^4–6^.

In the current study, we have successfully demonstrated a rapid, easy, feasible and reliable method to prepare mAb for IHC diagnosis by expressing part of protein after optimization in *E. coli* and immunization with Quickadjuvant.

## Electronic supplementary material

supplementary information
