# Supplementary material for: Preparation of Anti-Human Podoplanin Monoclonal Antibody and its application in Immunohistochemical Diagnosis
Source: Sci Rep. 2018 Jul 5;8:10162. doi: 10.1038/s41598-018-28549-w (PMC6033854; doi:10.1038/s41598-018-28549-w)
Supplement: Supplementary file 1 — supplementary information [file 41598_2018_28549_MOESM1_ESM.pdf]

Preparation of Anti-Human Podoplanin Monoclonal Antibody and its application in Immunohistochemical Diagnosis

Chengjie Xie[#1], Rongzhi Wang[#1],Abdullah F.U.H. Saeed[1], Qinghai Yang[2], Huiling Chen[2], Sumei Ling[1], Shiwei Xiao[1], Linmao Zeng[1], Shihua Wang[1,*]

[1]Key Laboratory of Pathogenic Fungi and Mycotoxins of Fujian Province, Key Laboratory of Biopesticide and Chemical Biology of Education Ministry, and School of Life Sciences, Fujian Agriculture and Forestry University, Fuzhou 350002, China
[2] Fuzhou Maixin Biotech.Co., Ltd, Fuzhou 350100 , China

*Correspondence:
Shihua Wang
wshyyl@sina.com

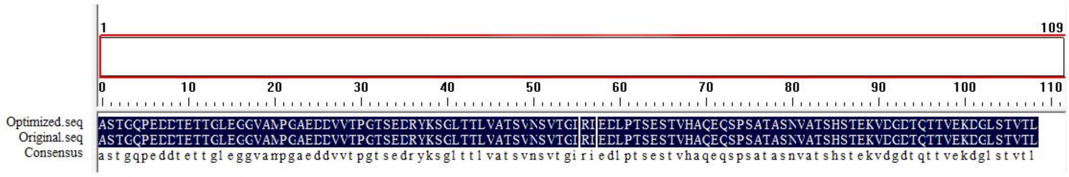

Fig S1. Sequence alignment of ePDPN between optimized sequence and original one. The optimized codon and the original one encode exactly the same protein sequence.
